# Interventions targeting social isolation in older people: a systematic review

**DOI:** 10.1186/1471-2458-11-647

**Published:** 2011-08-15

**Authors:** Andy P Dickens, Suzanne H Richards, Colin J Greaves, John L Campbell

**Affiliations:** 1 Primary Care Research Group, Peninsula College of Medicine & Dentistry, University of Exeter, Smeall Building, St Luke's Campus, Magdalen Road, Exeter, EX1 2LU, UK; 2 Institute of Health Service Research, Peninsula College of Medicine & Dentistry, University of Exeter, Veysey Building, Salmon Pool Lane, Exeter, EX2 4SG, UK

## Abstract

**Background:**

Targeting social isolation in older people is a growing public health concern. The proportion of older people in society has increased in recent decades, and it is estimated that approximately 25% of the population will be aged 60 or above within the next 20 to 40 years. Social isolation is prevalent amongst older people and evidence indicates the detrimental effect that it can have on health and wellbeing. The aim of this review was to assess the effectiveness of interventions designed to alleviate social isolation and loneliness in older people.

**Methods:**

Relevant electronic databases (MEDLINE, EMBASE, ASSIA, IBSS, PsycINFO, PubMed, DARE, Social Care Online, the Cochrane Library and CINAHL) were systematically searched using an extensive search strategy, for randomised controlled trials and quasi-experimental studies published in English before May 2009. Additional articles were identified through citation tracking. Studies were included if they related to older people, if the intervention aimed to alleviate social isolation and loneliness, if intervention participants were compared against inactive controls and, if treatment effects were reported. Two independent reviewers extracted data using a standardised form. Narrative synthesis and vote-counting methods were used to summarise and interpret study data.

**Results:**

Thirty two studies were included in the review. There was evidence of substantial heterogeneity in the interventions delivered and the overall quality of included studies indicated a medium to high risk of bias. Across the three domains of social, mental and physical health, 79% of group-based interventions and 55% of one-to-one interventions reported at least one improved participant outcome. Over 80% of participatory interventions produced beneficial effects across the same domains, compared with 44% of those categorised as non-participatory. Of interventions categorised as having a theoretical basis, 87% reported beneficial effects across the three domains compared with 59% of interventions with no evident theoretical foundation. Regarding intervention type, 86% of those providing activities and 80% of those providing support resulted in improved participant outcomes, compared with 60% of home visiting and 25% of internet training interventions. Fifty eight percent of interventions that explicitly targeted socially isolated or lonely older people reported positive outcomes, compared with 80% of studies with no explicit targeting.

**Conclusions:**

More, well-conducted studies of the effectiveness of social interventions for alleviating social isolation are needed to improve the evidence base. However, it appeared that common characteristics of effective interventions were those developed within the context of a theoretical basis, and those offering social activity and/or support within a group format. Interventions in which older people are active participants also appeared more likely to be effective. Future interventions incorporating all of these characteristics may therefore be more successful in targeting social isolation in older people.

## Background

In the United Kingdom, people aged 60 or above currently account for approximately 20% of the population [1], and this proportion is expected to rise to 24% by 2030 [2]. In comparison, 11% of the world's population was aged 60 or above in 2007, rising to an estimated 22% by 2050 [3]. The ageing population has resulted in emphasis being placed on health status trends of older people and how these trends may change in future, due to the anticipated increased demand for health and social care services [4].

The concept of social isolation has been defined in various ways in academic literature. Many authors agree that it is a uni-dimensional concept referring to the lack of social integration [5-8]. However, this assumes that all social contacts have the same social value or importance [9]. Alternate definitions of social isolation incorporate both 'structural' and 'functional' social support [10,11]. Structural social support is an objective assessment of size and frequency, while functional social support is a subjective judgement of the quality or perceived value of emotional, instrumental and informational support provided by others [12]. The second definition of social isolation is therefore multi-dimensional, including both the minimal quantity and quality of social support. We adopted the latter definition for the purposes of our review.

Two terms often used interchangeably in academic literature are 'social isolation' and 'loneliness'. Loneliness is a subjective concept resulting from a perceived absence or loss of companionship [6,13-15]. Social loneliness refers to negative feelings resulting from the absence of meaningful relationships and social integration, whereas emotional loneliness refers to the perceived lack of an attachment figure or confidant [16]. The two forms of loneliness differ in potential duration, as the former may be alleviated through gaining new acquaintances while the latter can only be resolved by the formation of an intimate bond [17], which may take longer.

While social isolation concerns the lack of structural and functional social support, loneliness relates specifically to one's negative feelings about that situation [18]. Expanding the distinction further, while social isolation may be either voluntary or involuntary, loneliness is always involuntary [6,19].

A recent meta-analysis of 148 longitudinal studies (308,849 participants, mean age of 64 years) reported a 50% reduction in the likelihood of mortality for individuals with strong social relationships [20]. A limitation of this review is that 'strong social relationships' was a composite variable that combined conceptually distinct measures of an individual's social context (e.g. loneliness, social isolation etc). Notwithstanding this, the authors observed that the impact of social relationships on the risk of mortality is comparable with major, well-established risk factors such as smoking and alcohol consumption, and exceeds that of physical inactivity and obesity. Studies focusing specifically on the measurement of social isolation and health report similar relationships. For example, social isolation is associated with increased mortality [21], poor self-rated physical health [22] and increased susceptibility to dementia [23] in the general population of older people, and with the onset of disability among older males living alone [24]. In a recent study we found that social isolation was negatively associated with health status and health-related quality of life of older people [25].

Risk factors for social isolation in older people include a lack of access to private transport, minimal or no contact with friends and family, low morale and living alone [26-28]. The prevalence of social isolation amongst older people is substantial, estimated to be 7 - 17% depending on the definition and outcome measure used [14,27,29-31]. The related, but distinct concept of loneliness is reported to be experienced by approximately 40% of this age group [32-34]. The prevalence of social isolation, combined with the evidence that it can impact on an individual's health and wellbeing, supports the targeting of social isolation as an important public health issue. The development of strategies to increase older people's participation in society has been a major, cross-cutting component of recent UK government policies regarding the delivery of health and social care, as well as wider policies and reviews relating to social cohesion and the tackling of social inequalities [35-39]. However, while there is a substantial evidence base supporting the targeting of social isolation as a public health priority, as well as a clear policy agenda advocating the implementation of interventions to ameliorate its effects, there remains considerable uncertainty regarding the characteristics of interventions that are effective and cost-effective in achieving these goals.

Three previous systematic reviews [5,40,41] have synthesised the evidence of the effectiveness of interventions targeting social isolation in older people. Findlay [40] selected papers published between 1982 and 2002, while Cattan & White [41] included papers between 1970 and 1997, and Cattan et al [5] included papers published between 1970 and 2002. Experimental, quasi-experimental and before-and-after study designs were included. Each of these reviews focused on loneliness and social isolation as the main outcomes under investigation, as previous research had used the terms interchangeably [5].

A recent meta-analysis of 50 studies exclusively aimed at ameliorating feelings of loneliness [42] has been published since the date of our literature search. However, we contend that our review remains timely and relevant. Firstly, the scope of the review by Masi et al differs and their results - relating to the concept of loneliness - do not fully address the effectiveness of interventions aimed at tackling social isolation. In addition, the search strategy was relatively limited (combining six keywords on PubMed and PsycINFO), and may be insufficient to identify all relevant studies. The authors used meta-analysis to pool data from a heterogeneous sample of participants (e.g. school children, homeless youths, older people) and interventions (e.g. online chat rooms, physical exercise, social activities and support groups) studied. There is considerable academic debate regarding the appropriateness of meta-analysis in the context of such clinical heterogeneity [43,44]. Despite this, the pooling of data from such a wide range of contexts leaves much room for further explanation of mechanisms under-pinning the observed variations in effectiveness.

Given the lack of consensus regarding the definition of social isolation, previous reviews may not have identified all relevant intervention effects. According to the multi-dimensional definition of social isolation used in our review, outcomes regarding structural social support and functional social support are important indicators of effect. In addition, reporting on mental and physical health outcomes known to be associated with both social isolation and loneliness may also contribute to the understanding of intervention effects. The need to consider a broader range of participant outcomes, combined with the substantial body of research relating to social isolation published since 2002, supports the need for an updated review.

### Research question

To determine the effectiveness of interventions designed to alleviate social isolation and/or loneliness in older people, we reviewed randomised controlled trials and quasi-experimental studies that assessed treatment effects of such interventions, in comparison with inactive controls. A secondary objective was to identify the potential health benefits of such interventions.

## Methods

Systematic reviews represent a scientific synthesis of evidence using reproducible and pre-determined methods [45]. Literature searches were conducted on the following electronic databases, for studies published before May 2009: MEDLINE, EMBASE, ASSIA, IBSS, PsycINFO, PubMed, DARE, Social Care Online, the Cochrane Library and CINAHL. Search criteria (Table 1) were tailored according to the database. Citation tracking was used to identify additional studies from the reference lists of previous relevant systematic reviews. Potentially eligible studies were identified by a reviewer (AD) scanning titles and abstracts. Where there was uncertainty about potential eligibility, a second reviewer (SR or CG) read the abstracts allowing a joint decision to be made. Full text papers of all potentially eligible studies were obtained to enable data extraction.

**Table 1:** Search strategy

1.	befriend$. ti, ab.
2.	(home adj visit$). ti, ab.
3.	(visit$ adj program$). ti, ab.
4.	mentor$3. ti, ab, kw.
5.	Mentors/
6.	4 not 5
7.	psychosocial. ti, ab.
8.	network$3. ti, ab, kw.
9.	prevent$3. ti, ab.
10.	promot$. ti, ab.
11.	support. ti, ab, kw.
12.	self-help. ti, ab, kw.
13.	(self adj help). ti, ab, kw.
14.	(social$2 adj activ$5). ti, ab.
15.	Health Promotion/
16.	(health adj promotion). ti, ab, kw.
17.	(health adj status). ti, ab, kw.
18.	exp community networks/
19.	(social$2 adj participat$3). ti, ab.
20.	(social$2 adj integrat$3). ti, ab.
21.	friendship. ti, ab, kw.
22.	(quality adj2 life). ti, ab.
23.	(well adj being). ti, ab, kw.
24.	wellbeing. ti, ab, kw.
25.	(self adj esteem). ti, ab.
26.	exp Self Esteem/
27.	(creative adj activ$5). ti, ab, kw.
28.	exercise. ti, ab, kw.
29.	(physical adj activ$5). ti, ab, kw.
30.	peer. ti, ab, kw.
31.	socio-medical. ti, ab, kw.
32.	1 or 2 or 3 or 6 or 7 or 8 or 9 or 10 or 11 or 12 or 13 or 14 or 15 or 16 or 17 or 18 or 19 or 20 or 21 or 22 or 23 or 24 or 25 or 26 or 27 or 28 or 29 or 30 or 31
33.	Middle Aged/
34.	Aged/
35.	"Aged, 80 and over"/
36.	geriatric. ti, ab.
37.	elder$2. ti, ab, kw.
38.	older$. ti, ab, kw.
39.	senior$. ti, ab.
40.	(ageing or aging). ti, ab, kw.
41.	(old$2 adj age$3). ti, ab.
42.	aged. ti, ab.
43.	retire$4. ti, ab.
44.	33 or 34 or 35 or 36 or 37 or 38 or 39 or 40 or 41 or 42 or 43
45.	(social$2 adj4 isolat$3). ti, ab, kw.
46.	(isolated adj (elder$ or old$)). ti, ab.
47.	(social$2 adj alienat$3). ti, ab.
48.	(social$2 adj exclu$). ti, ab, kw.
49.	(social adj contact$). ti, ab.
50.	(social adj environment). ti, ab, kw.
51.	lonel$5. ti, ab, kw.
52.	loss. kw.
53.	bereave$4. kw.
54.	Loneliness/
55.	Social Isolation/
56.	Social Alienation/
57.	Social Distance/
58.	45 or 46 or 47 or 48 or 49 or 50 or 51 or 52 or 53 or 54 or 55 or 56 or 57
59.	Intervention Studies/
60.	intervention. ti, ab.
61.	Program Evaluation/
62.	program$2 evaluation"tabcaption". ti, ab.
63.	59 or 60 or 61 or 62
64.	exp randomized controlled trials/
65.	"randomized controlled trial". pt.
66.	"controlled clinical trial". pt.
67.	(random$ or placebo$). ti, ab, sh.
68.	((singl$ or double$ or triple$ or treble$) and (blind$ or mask$)). tw, sh.
69.	(retraction of publication or retracted publication). pt.
70.	64 or 65 or 66 or 67 or 68 or 69
71.	(animals not humans). sh.
72.	70 not 71
73.	exp case-control studies/
74.	controlled clinical trial/
75.	exp clinical trial/
76.	control$3. ti, ab.
77.	(quasi adj experiment$2). ti, ab.
78.	match$3. ti, ab.
79.	trial. ti, ab.
80.	(controlled adj5 trial). ti, ab, kw.
81.	(randomi?ed adj controlled adj trial). ti, ab.
82.	63 or 72 or 73 or 74 or 75 or 76 or 77 or 78 or 79 or 80 or 81
83.	32 and 44 and 58 and 82

### Study selection

Studies were eligible for inclusion if they met the following criteria:

• related in full/part to older people;

• the intervention targeted people identified as socially isolated and/or lonely, and stated a clear and plausible aim to alleviate this;

• recorded some form of participant-level outcome measure, and reported sufficient outcome data for treatment effects to be obtained;

• used a randomised controlled trial (RCT), or quasi-experimental (controlled trial or matched controlled trial) design;

• included an inactive (usual care, no intervention, attentional) control group;

• was published in English.

Consistent with the aim of reviewing the effectiveness of interventions, comparative, experimental studies were selected. Both RCTs and quasi-experimental designs, comparing the intervention with a control group, were eligible for inclusion to maximise the number of included studies. Evidence suggests that restricting eligibility to RCTs may be unhelpful [46], particularly within health promotion and public health contexts where experimental designs are often not possible or feasible. Study eligibility for inclusion in the review was assessed by two reviewers (AD and SR or CG), with disagreements being resolved by consensus.

The studies were categorised as having a theoretical basis if they cited a specific theory underlying their intervention design, or reported that the intervention was based on a broad theoretical approach.

### Quality assessment of included studies

The first reviewer (AD) rated the quality of randomised and non-randomised studies according to the Cochrane risk of bias tool and the Newcastle-Ottawa Scale respectively.

The Cochrane risk of bias tool [47] includes six domains ('random sequence generation', 'random allocation concealment', 'blinding', 'incomplete outcome data', 'selection outcome reporting', and 'other sources of bias'), each of which were completed according to whether they had been addressed in the study (yes/no/unclear). We added an extra domain regarding whether the analysis had been adjusted for baseline imbalances (where appropriate). Although not affecting the inclusion or exclusion of studies, the above domains were used to generate an overall quality score. Two domains, sequence generation and incomplete data, were prioritised as non-representative samples and high loss to follow-up rates are considered major threats to the external validity of community-based studies. An overall risk of bias score of 'high', 'moderate' or 'low' was generated for each study using the score for the two prioritised domains. A high risk of bias score was recorded if either of these prioritised domains were not addressed, or if both domains were unclear. Moderate risk of bias scores reflected one prioritised domain being addressed while the other was unclear, and low risk of bias scores reflected both prioritised domains being addressed.

The Newcastle-Ottawa Scale (NOS) for cohort studies [48] was used to assess the quality of non-randomised trials. The NOS allocates points for three domains of 'selection', 'comparability' and 'outcome' (maximum score = 9). As this review included controlled trials rather than cohort studies, we modified the NOS by excluding the item relating to baseline exposure status, yielding a maximum possible score of 8 points. The risk of bias was then categorised as high (0 to 3 points), moderate (4 or 5) or low (6 to 8).

### Data extraction, analysis and synthesis

Two reviewers (AD and SR or CG) independently extracted data from the eligible studies using a standardised checklist. Data were extracted for three outcome domains including social health (four sub-domains of: 'loneliness', 'social isolation', 'structural social support', 'functional social support'); mental health (two sub-domains of: 'depression', 'mental/psychological wellbeing') and physical health (e.g. perceived health status, blood pressure, daily medication intake). Social health and mental health data were extracted as between-group differences with 95% confidence intervals and p values. Physical health data were extracted as p values and direction of effect, due to the wide variety of outcomes assessed.

Data relating to the outcome domains of social, mental and physical health were collected using both validated and non-validated outcome measures (see Table 2 for the specific tools considered in our review). Validated outcome measures were defined as those supported by an academic reference and evidence of their psychometric properties. Instances of authors using selected items, rather than the full scales of validated measures led to measures being categorised as 'partially validated'. Non-validated outcome measures were those developed by the authors for the purposes of the study.

**Table 2:** Characteristics of studies stratified by i) study design and ii) delivery mode

Study, year (country)	Participants	Explicit targeting	Activity	Groups		Outcomes considered in the review
					
				Intervention	Control	
**RANDOMISED CONTROLLED TRIALS (n = 16):**						

**Group**						

Constantino, 1988 (USA) [66]	Community-dwelling, widows	No	Support group	(1) Bereavement crisis intervention (BCI). Set in University. Weekly 1.5 hr planned group discussions on set themes. Six weeks. (2) Social adjustment intervention (SAI). Set in venue of selected activities. Weekly activities for 6 weeks.	Not described	Revised Social Adjustment Scale (RSAS), Beck Depression Inventory (BDI), Depression Adjective Check List (DACL) Form E

Fukui et al, 2003 (Japan) [55]	Women with primary breast cancer	No	Education/support group	Psychosocial group inc. health education, coping skills, stress management, set in hospital. Three groups of 6-10 patients, weekly 1.5 hrs meeting for 6 weeks.	Waiting list control	Revised UCLA Loneliness Scale, Utilisation of confidants questionnaire, Satisfaction with mutual aid with other cancer survivors

Harris & Bodden, 1978 (USA) [67]	Community-dwelling, Meals on wheels recipients	Yes	Social activity	Activity group, setting not stated. 1 × weekly 2 hrs session, for 6 weeks.	Usual care	Shortened 35-item version of Chicago Activity Inventory

Kremers et al, 2006 (Netherlands) [68]	Community-dwelling, single women	No	Self-management group	Self-management group intervention, setting not reported. 6 × 2.5 hr weekly meetings.	No intervention	de Jong Gierveld Loneliness Scale, Social Production Function Index Level Scale

Lokk, 1990 (Sweden) [69]	Community-dwelling, people with handicaps	No	Discussion group	Group discussion re: goals plus standard reactivation programme, set in day care centre. Sessions twice a week (?12 weeks) inc. discussion, feedback and decision making.	Usual care - standard reactivation programme, set in day care centre	Activities outside institution, social network index, contact desire index, Hopelessness Index, Depression Index, loneliness, perceived health

Ollonqvist et al, 2008 (Finland) [70]	Community-dwelling, at risk of institutionalisation within 2 yrs due to decreasing functional capacity	No	Physical activity	Inpatient geriatric rehabilitation, including group physical activities, group discussions and lectures. Based at rehab centre. Eight months duration.	No intervention	Loneliness, Loneliness causing insecurity, Being left alone causing insecurity, Satisfaction with engagement with their children, Number of friends and relatives, Geriatric Depression Scale (GDS-15)

Routasalo et al, 2009 (Finland) [13]	Community-dwelling, reported feelings of loneliness	Yes	Social activity	Psychosocial group nursing, inc art & inspiring activities, exercise & discussions, therapeutic writing & group therapy. Based in community centres. Weekly sessions over 3 month period.	No intervention	UCLA Loneliness Scale (version 3), Lubben's Social Network Scale, Social activity, psychological wellbeing

Savelkoul & de Witte (2004) (Netherlands) [56]	Chronic rheumatic disorder patients	Yes	Coping education group	Coping group, unsure of setting. Groups of 10-12 pts, 10 × 2 hr sessions over 13 weeks. Awareness raising of social support sources.	(1) Mutual support group, unsure of setting. 5 × groups of 10-12 patients, 10 × 2 hr sessions over 13 weeks. (2) Waiting list control	Social Support List-Interactions, de Jong Gierveld Loneliness Scale, Sickness Impact Profile 68

White et al, 2002 (USA) [64]	Nursing home and congregate housing residents	No	Internet training	Internet training, set in nursing homes/congregate housing. 9 hrs group training over 2 weeks. 24 hr access to computers, for 5 months.	Usual care, nursing homes/congregate housing.	Modified form of revised UCLA Loneliness scale for use with older adults, Number of confidants in their life, CES-Depression scale

**One-to-one**						

Brennan et al, 1995 (USA) [51]	Community-dwelling, Caregivers of Alzheimer's Disease (AD) sufferers	No	Computer support network	Provision of & training for, a computer network for AD caregivers. Set in ppts' homes. 90 mins training session. 24 hr access to software for 12 months. Monthly phone calls on service use.	90 mins training session, identifying local services & resources. Monthly phone calls on service use, for 12 months	Instrumental and Expressive Social Support Scale, Centre for Epidemiological Studies Depression scale, Contact with community and medical services

Heller et al, 1991 (USA) [58]	Low-income housing residents	Yes	Telephone support	(1) Staff telephone contact & peer telephone dyads (as initiator), ppts' home. Frequency not reported. 30 week duration. (2) As above, but was recipient in peer telephone dyad. (3) Staff telephone contact only, ppts' home. Frequency not reported. 20 week duration. (4) Staff telephone contact. 10 week duration.	No intervention	Paloutzian & Ellison Loneliness Scale, Perceived Social Support Scale, Network embeddedness, Philadelphia Geriatric Center Morale Scale, Center for Epidemiological Studies Depression Scale

MacIntyre et al, 1999 (Canada) [71]	Recipients of home nursing & homemaking services	Yes	Home visiting	Volunteer visitor programme, clients' home. Weekly 3 hr visits, for 6 weeks. Activities were mutually agreed.	Usual care control group	Personal Resource Questionnaire

O'Loughlin et al, 1989 (Canada) [57]	Chronic mental health problems, socially isolated	Yes	Home visiting	Volunteer visiting, in clients' home. Weekly visits, duration not reported. Provision of info re: medical/community resources.	Waiting list control	Recent social and leisure activities

Schulz, 1976 (USA) [59]	Private, church-affiliated retirement home residents	No	Home visiting	(1) Friendly visiting, set in retirement home. Ppts controlled duration and frequency of visits. Two-month visiting period. (2) Friendly visiting, predicted but not controlled by ppt, set in retirement home. Two-months visiting period.	(1) Random friendly visits, set in retirement home. No notification given of visits. Two-month visiting period. (2) No visit comparison group	Activity index, % of time per day spent in active pursuits, % of next 7 days devoted to special commitments, Tri scale activity composite, Wohlford hope scale, happiness, medication taken/day

Slegers et al, 2008 (Netherlands) [72]	Community-dwelling, no prior computer experience	No	Computer/internet training	Computer & internet training, provision of PC. 3 × 4 hr training sessions over 2-week period. PC use for 12 months.	(1) Not interested. No PC use for 12 months (2) Interested, but no training. No PC use for 12 months (3) Interested, received training. No PC use for 12 months	de Jong Gierveld Loneliness Scale, social networks, SF-36 Mental Component Summary, depression subscale of Symptoms Check List, anxiety subscale of Symptoms Check List

**Mixed mode**						

Drentea et al, 2006 (USA) [52]	Caregivers to Alzheimer's disease (AD) sufferers	No	Counselling/support group	Individual & family counselling, support group and ad hoc counselling. Setting unclear. Four months duration. Subsequent attendance at support groups and contact with counsellors for up to five years.	Usual care. Received resources information pack and referrals on request	Items from the Stokes Social Network List, Satisfaction with social support

**QUASI-EXPERIMENTAL STUDIES (n = 16):**						

**Group**						

Arnetz & Theorell, 1983 (Sweden) [60]	Senior citizen apartment residents	No	Social activity	Activity programme, set in apartment. Assistance to organise social activity groups & outings. 4-8 ppts per group, met once or twice a week for 6 months.	No intervention	Participation in bureau/church/occupational therapy activities, depression, suicidal thoughts.

Baumgarten et al, 1988 (Canada) [61]	Residents of two govt subsidised apartment buildings	Yes	Social activity	Activity group including a mutual help network and leisure/cultural group activities. Set in apartment building. 11 month duration.	No intervention	Number of social ties, Index of support satisfaction, Center for Epidemiologic Studies Depression

Evans & Jaureguy, 1982 (USA) [73]	Blind, community-dwelling	No	Group therapy	Phone group therapy. Set at home. 8 × 1 hr weekly phone conference calls. Eight week duration.	No intervention	UCLA Loneliness Scale, Wakefield self-rating depression scale, Outside social activities, Household chores

Fujiwara et al, 2009 (Japan) [74]	Community-dwelling	No	Social activity	Picture book reading to children, set in schools. Weekly or bi-weekly school visits for 18 months. Mutual learning monthly meetings.	Conventional social activities, setting not specified	Social activity checklist, Social networks, Social support scale

Martina & Stevens, 2006 (Netherlands) [75]	Participants of a Friendship Programme for older women (int) & community-dwelling (control)	Yes	Educational programme	Educational friendship programme, setting not reported. 12 lessons (duration of lessons & programme not reported).	No intervention	de Jong Gierveld Loneliness Scale, items from the Assertiveness scale, Personal Convoy Model of relationships, Positive and Negative Affect Scale

Rosen & Rosen, 1982 (USA) [76]	Community-dwelling, member of senior citizen centre	No	Group counselling	Mental health counselling group, set in local senior centres. Group meetings (?2 hrs, ?weekly), met for 40-49 sessions over 12-15 months.	(1) Comparison group, not needing MH services (2) Control group, in need of MH services	Social isolation, activity and morale measures from OARS

Stevens & van Tilburg, 2000 (Netherlands) [77]	Participants of a Friendship Programme for older women (int), community-dwelling (control)	Yes	Educational programme	Educational friendship programme, setting not reported. 12 lessons (duration of lessons & programme not reported).	No intervention	de Jong Gierveld Loneliness Scale

Toseland et al, 1990 (USA) [53]	Caregivers, community-dwelling	No	Support group	(1) Group support. Setting not stated. 8 × weekly 2 hr sessions. (2) Individual counselling. Setting not stated. 8 × weekly 1 hr sessions.	Control group. Given funding for respite, community resources information & a referral to a community agency if requested	Change in support network size, Extent of support, Satisfaction with support network, Bradburn Affect Balance Scale, Brief Symptom Inventory

White et al, 1999 (USA) [62]	Retirement community residents	No	Internet training	Internet training set in retirement community. Nine hrs training. 24 hr access to PCs. Five month duration.	Comparison group. No PC use during the study. Offered computer training after study	UCLA Loneliness Scale, Duke Social Support Index, Bradburn Affect Balance Scale, CES-Depression scale

Winningham & Pike, 2007 (USA) [63]	Assisted Living Facility (ALF) residents	No	Cognitive behavioural therapy	Cognitive Enhancement Programme, in ppts' ALF. 3 × sessions per week. Three month duration.	Usual care control group	Self-appraisal re: their social support, beliefs re: family/friends support behaviour, UCLA Loneliness Scale v3

**One-to-one**						

Bogat & Jason, 1983 (USA) [78]	Community-dwelling, on waiting list for a Friendly Visitor Programme	Yes	Home visiting	(1) Network-building visiting programme, set in clients' home. Weekly, 1 hr visits for 3 months. (2) Relationship-oriented visiting programme, set in clients' home. Weekly, 1 hr visits for 3 months	No intervention	Current networks, desired networks, N phone calls/week, N visitors/visits made per week

Fokkema & Knipscheer, 2007 (Netherlands) [79]	Community-dwelling, living alone, lonely	Yes	Internet training	Internet training, set in clients' home. Internet access for three years. 5 × 2 hr lessons & home visits every two or three weeks.	No intervention	de Jong Gierveld Loneliness Scale inc. social & emotional loneliness subscales

Mulligan & Bennett, 1977 (USA) [80]	Community-dwelling, very isolated	Yes	Home visiting	Friendly visiting programme, ppts' homes. 1 × 1 hr structured home visit every two weeks, for six months.	No intervention	Past Month Isolation Index, Mental Status Questionnaire, Mental Status Schedule

Rook & Sorkin, 2003 (USA) [81]	Community-dwelling attenders of regional centres for lower-income older adults	No	Social activity	Foster Grandparent Programme for developmentally-disabled child, set at hospital. Contact with child 4 hrs/day, five mornings a week. Duration not reported.	(1) non-volunteer programme with access to age peers. Content not reported. (2) not reported - assuming no intervention	Abbreviated UCLA Loneliness Scale, number of new relationships formed in past year, number of people who depended on the participant, Center for Epidemiological Studies-Depression Scale, Rosenberg Self-Esteem Scale

Toseland & Smith, 1990 (USA)[54]	Caregivers, community-dwelling	No	Counselling	(1) Individual professional counselling. Setting not stated. 8 × weekly 1 hr sessions. (2) Individual peer counselling. Setting not stated. 8 × weekly 1 hr sessions.	No intervention	Number of people in network, Change in support network, Bradburn Affect Balance Scale, Brief Symptom Inventory,

**Service provision**						

Bergman-Evans, 2004 (USA) [65]	Residents from two types of nursing home	No	Service provision	Human Habitat model of care, set in nursing home. Daily contact with pets, plants and children. One year duration.	Standard nursing home model (non-profit). Usual care	UCLA Loneliness Scale (version 3), helplessness item of GDS-30, boredom item of GDS-30

Due to the heterogeneity of both the interventions studied and the outcome data extracted, quantitative synthesis of the data using meta-analytical techniques was deemed inappropriate [43]. Narrative synthesis was conducted to summarise the effectiveness of interventions. To aid interpretation of the substantial number of studies identified, we also adopted an approach similar to vote-counting [49], whereby we categorised intervention effects as 'significantly beneficial' or 'not beneficial'.

The systematic review was reported according to the PRISMA Statement [50].

## Results

### Study selection

**Figure 1:**
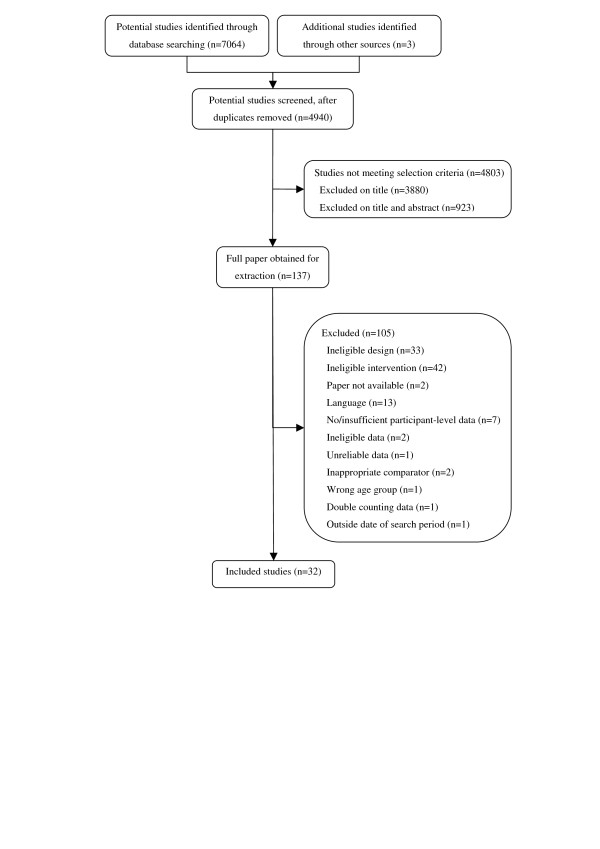
**Eligibility and screening of papers considered for the systematic review**.

Of the 7067 studies identified, 6930 did not meet the selection criteria. Full papers were obtained for 137 studies. After application of the study inclusion criteria, 32 studies were deemed eligible for inclusion (Figure 1).

### Description of included studies

Sixteen RCTs and 16 quasi-experimental studies were included (Table 2). A total of 4061 participants contributed to the 32 studies, with between 23 and 741 participants per study. Participants (Table 2) included caregivers [51-54], disease sufferers [55-57], housing residents [58-63], residents in institutional settings [64,65] and community-dwelling older people [13,66-81]. Only 12/32 (38%) studies explicitly targeted people identified as being socially isolated or lonely (Table 2) via study assessment or prior self- or professional-assessment [13,56-58,61,67,71,75,77-80]. The remaining studies targeted people for whom social isolation and loneliness was implied or assumed based on personal circumstance, such as being a resident in a nursing home or a care giver.

Table 2 describes the type of intervention evaluated and control group employed, as well as the outcome measures synthesised in this review. Nineteen interventions were delivered in groups [13,53,55,56,60-64,66-70,73-77], 11 were delivered one-to-one [51,54,57-59,71,72,78-81], one used a combination of group and one-to-one formats [52] and one was concerned with the provision of an alternative form of nursing home care [65].

The frequency of contact varied, from those delivered as individually tailored [52] interventions, to the majority that were delivered on a weekly or fortnightly basis (one intervention entailed participant contact three times per week [63]). Most interventions lasted between six weeks and one year, with several lasting up to three [79] and five [52] years. Some studies omitted details of the frequency [58,75,77] or duration [57,75,77,81] of the intervention.

Interventions were delivered by health or social care professionals [13,51,52,56,60,61,65,69,70,76,81], instructors [62,64,72,79], students [59,66,71,78], counsellors [53,54,73] and unspecified professionals [55,57,58,68,74,80]. Four studies [63,67,75,77] did not report who delivered the interventions.

Studies reported a range of comparators including: no intervention [13,54,58,60-62,66,68,70,73,75,77-80], usual care [52,63-65,67,69,71,74], waiting list [55,57], and attentional [51,53] control groups. Five studies used multiple comparators [56,59,72,76,81].

Interventions were categorised as offering activities (social or physical programmes), support (discussion, counselling, therapy or education), internet training, home visiting or service provision (Table 2). Seven studies evaluated activity interventions [13,60,61,67,70,74,81], fifteen evaluated support interventions [51-56,58,63,66,68,69,73,75-77], five evaluated home visiting [57,59,71,78,80], four evaluated internet training interventions [62,64,72,79], and one evaluated a service provision intervention [65].

Interventions were further defined as 'participatory' or 'non-participatory', depending on whether they entailed active input from participants involving social contact (not necessarily face to face) rather than them simply being recipients of a service or education/training. Twenty three studies were participatory [13,51-56,58,60,61,63,65-70,73-77,81] and the remaining nine were non-participatory [57,59,62,64,71,72,78-80].

Eighteen studies had only one follow-up, conducted between six weeks and three years after the baseline assessment [51,53,54,59,61-65,67,70,71,73,74,76-79]. The remaining 14 studies consisted of multiple follow-up points, of which 13 had between two and four follow-up points covering periods of between six months and two years [13,55-58,60,66,68,69,72,75,80,81], while the final study [52] involved 11 assessments over a five year period.

### Methodological quality

Ten out of the 16 RCT studies included in this review were judged to be at moderate risk of bias [13,51,55-57,64,66,70-72] (Table 3), compared with only one of the 16 quasi-experimental studies [61] (Table 4). The remaining six RCT and 15 quasi-experimental studies were judged to be at high risk of bias. Tables 3 and 4 includes description of the methodological properties of included studies that were used to generated risk of bias scores (RCT: sequence generation and/or loss to follow-up; quasi-experimental: total number of quality criteria addressed).

**Table 3:** Quality of RCT studies included in the systematic review

Study	**Adequate sequence generation**^**a**^	**Allocation concealment**^**a**^	**Blinding**^**a**^	**Incomplete data addressed**^**a**^	**Free of selective reporting**^**a**^	**Adjusted for imbalance at baseline**^**a**^	**Free of other bias**^**a**^	Risk of bias
Constantino [66]	Yes	No	Unclear	Unclear	Yes	Yes	Yes	Moderate

Fukui et al [55]	Unclear	Unclear	Unclear	Yes	Yes	Yes	Yes	Moderate

Harris & Bodden [67]	Unclear	Unclear	Unclear	Unclear	Yes	Yes	Unclear	High

Kremers et al [68]	Unclear	Unclear	Unclear	Unclear	Yes	Yes	Yes	High

Lokk [69]	Unclear	Unclear	Unclear	Unclear	Yes	Yes	Yes	High

Ollonqvist et al [70]	Yes	Yes	Yes	Unclear	Yes	Yes	Yes	Moderate

Routasalo et al [13]	Yes	Yes	Unclear	Unclear	Yes	Yes	Yes	Moderate

Savelkoul & de Witte [56]	Unclear	Yes	Yes	Yes	Yes	Yes	Yes	Moderate

White et al, 2002 [64]	Unclear	Unclear	Unclear	Yes	Yes	Yes	Unclear	Moderate

Brennan et al [51]	Unclear	Unclear	No	Yes	Yes	Yes	Yes	Moderate

Heller et al [58]	Unclear	Unclear	Unclear	Unclear	Yes	Yes	Yes	High

MacIntyre et al [71]	Unclear	Unclear	Unclear	Yes	Yes	Yes	Unclear	Moderate

O'Loughlin et al [57]	Yes	Yes	Yes	Unclear	Unclear	Yes	Yes	Moderate

Schulz [59]	Unclear	Unclear	Unclear	Unclear	No	Yes	No	High

Slegers et al [72]	No	Unclear	Unclear	Yes	Yes	Yes	No	Moderate

Drentea et al [52]	Unclear	Unclear	Unclear	Unclear	Yes	Yes	Yes	High

^a ^Refer to Higgins & Altman [47] for a definition of these categories

**Table 4:** Quality of quasi-experimental studies included in the systematic review

Study	**Selection**^**a**^					**Comparability**^**a**^		**Outcome**^**a**^					Risk of bias
	**1a)** **Int. group truly representative**	**1b)** **Int. group somewhat representative**	**2a)** **Cont. group from same community**	**3a)** **Secure record used**	**3b)** **Structured interview used**	**1a)** **Important factor controlled**	**1b)** **Additional factor controlled**	**1a)** **Blind assessment**	**1b)** **Record linkage**	**2a)** **Sufficient follow-up period**	**3a)** **Complete follow-up**	**3b)** **Attrition unlikely to affect bias**	

Arnetz & Theorell [60]	No	Yes	Yes	No	No	No	No	No	No	Yes	No	No	High

Baumgarten et al [61]	No	No	No	No	Yes	Yes	Yes	No	No	Yes	No	No	Moderate

Evans & Jaureguy [73]	No	Yes	Yes	No	No	No	No	No	No	Yes	No	No	High

Fujiwara et al [74]	No	No	Yes	No	No	No	No	No	No	Yes	No	No	High

Martina & Stevens [75]	No	No	No	No	No	No	No	No	No	Yes	No	Yes	High

Rosen & Rosen [76]	No	No	No	Yes	No	No	No	No	No	Yes	No	No	High

Stevens & van Tilburg [77]	No	No	No	No	No	Yes	Yes	No	No	Yes	No	No	High

Toseland et al [53]	No	No	Yes	No	No	No	No	No	No	Yes	No	Yes	High

White et al 1999 [62]	No	No	Yes	No	No	No	No	No	No	Yes	No	No	High

Winningham & Pike [63]	No	No	No	No	No	No	No	No	No	Yes	No	No	High

Bogat & Jason [78]	No	No	No	No	No	Yes	No	No	No	Yes	No	No	High

Fokkema & Knipscheer [79]	No	No	No	No	No	No	No	No	No	Yes	No	No	High

Mulligan & Bennett [80]	No	No	Yes	No	No	No	No	No	No	Yes	No	No	High

Rook & Sorkin [81]	No	No	No	No	No	Yes	Yes	No	No	Yes	No	No	High

Toseland & Smith [54]	No	No	No	No	No	No	No	No	No	Yes	No	Yes	High

Bergman-Evans [65]	No	No	No	No	No	No	No	No	No	Yes	No	No	High

^a ^Refer to Wells et al [48] for full description of these categories

Poor reporting of analyses was evident particularly when reporting a lack of intervention effect, including the absence of significance values [13,54,57,58,76,81] and participant-level outcome data [13,56] for some outcome measures. Only two studies [62,70] identified a primary outcome measure and only one study [70] reported a sample size calculation. Hence for the most part it was not possible to conclude whether the studies had sufficient power to detect statistically significant differences.

Twenty two out of the 32 included studies [13,51,55,56,58,59,62,63,65-68,71-77,79-81] used validated outcome measures, and three studies [52,57,64] used partially validated measures to assess the social health outcomes of loneliness, social isolation, structural social support or functional social support. Nineteen out of the 21 studies assessing mental health (depression or mental/psychological wellbeing) used validated outcome measures [51,53,54,56,58,59,61,62,64,66,68-70,72,73,75,76,80,81], and one [65] used a partially validated measure. Of the five studies assessing physical health, only one [56] used a validated outcome measure.

### Study findings regarding effectiveness

We present study data according to the mode of intervention delivery (group, one-to-one, service delivery) and intervention type (activity, support, internet training, home visiting, service provision), stratified by risk of bias but not by study design. For brevity, we discuss only the studies where a significant improvement was found in at least one of the three outcome domains extracted. Studies with non-significant results are not discussed, although their data are presented in the supporting tables. Table 5 summarises the study findings for the four social health outcomes of loneliness, social isolation, structural social support and functional social support, stratified by study design. Table 6 presents the results of the vote counting process adopted for the three outcome domains of social, mental and physical health.

**Table 5:** Study results for outcomes of loneliness, social isolation, structural social support and functional social support

Study	N (n allocated to intervention, control)	Mean age (yrs)	Gender (% female)	Intervention effect on loneliness/social isolation/structural social support/functional social support
**RANDOMISED CONTROLLED TRIALS (n = 16):**				

Constantino [66]	150 (i1 = 50, i2 = 50, c = 50)	57.98 overall	100% (i1) 100% (i2) 100% (c)	Improved socialisation across 12 months. Most notable at 6 weeks but remained better than control at all time points.

Fukui et al [55]	50 (25, 25)	53.5 overall 52.6 (i) 54.3 (c)	Not reported	At six week and six months, reduced loneliness and increased number of confidants. Improved satisfaction with confidants and mutual aid at both time points.

Harris & Bodden [67]	102 (i = 26, c1 = 26)	76.6 overall Not reported (i) Not reported (c)	Not reported	Increased social interaction at six weeks.

Kremers et al [68]	142 (63, 79)	Overall NR 62.8 (i) 65.2 (c)	100%	No effect on overall or emotional loneliness at six weeks and six months. Reduced social loneliness at six weeks, but effect disappeared at six months.

Lokk [69]	65 (33, 32)	Overall NR 76 (new i), 78 (chronic i) 74 (new i), 71 (chronic i)	Overall NR 52% (new i), 50% (chronic i) 42 (new i), 63 (chronic i)	Increased social network size at six weeks, but effect disappeared by 12 weeks. Increased availability of company evident at 24 weeks.

Ollonqvist et al [70]	741 (376, 365)	78 overall 78.1 (i) 78.6 (c)	86% overall 84.6 (i) 87.1 (c)	No effect on loneliness or number of friends and relatives at 12 months.

Routasalo et al [13]	235 (117, 118)	Overall NR 80 (i) 80 (c)	Overall NR 74.4 (i) 72.9 (c)	Developed more new friendships at 12 months.

Savelkoul & de Witte [56]	168 overall (i = 56, c1 = 56, c2 = 56)	Overall NR 52.5 (i) 51.5 (c1) 50.5 (c2)	Overall NR 76.8% (i) 58.9% (c1) 77.9% (c2)	No effect on loneliness at post-intervention or six months.

White et al, 2002 [64]	100 (51, 49)	Overall NR 71 (i) 72 (c)	Overall NR 71 (i) 82 (c)	No effect on loneliness or number of confidants at five months.

Brennan et al [51]	102 (51, 51)	64 overall	67% overall	No effect on social isolation at 12 months.

Heller et al [58]	291 (238 - not split by grp, 53)	74 overall^a^	100% overall	None of the intervention groups reported an effect on loneliness or perceived level of support from friends and family members at 20 or 30 weeks.

MacIntyre et al [71]	26 (15, 11)	79.4 overall 79.7 (i) 79.0 (c)	68% overall 58% (i) 80% (c)	Increased social integration at six weeks, but no effect on perceptions of intimacy, nurturance and guidance.

O'Loughlin et al [57]	74 (39, 35)	42 overall 42.6 (i) 41.2 (c)	Overall NR 46.2 (i) 57.1 (c)	No effect on recent social and leisure activities, or satisfaction with social relationships at either three or six months.

Schulz [59]	40 (i1 = 10, i2 = 10, c1 = 10, c2 = 10)	81.5 overall 85.0 (i1) 79.8 (i2) 83.4 (c1) 77.9 (c2)	90% overall	At two months, increased social activity, amount of time spent in active pursuits and number of activities planned. Intervention effects were only significant when comparing both intervention groups against both control groups.

Slegers et al [72]	236 (i = 62, c1 = 45, c2 = 68, c3 = 61)	Not reported	Not reported	No effect on loneliness or social network size at either four or 12 months compared with all three control groups.

Drentea et al [52]	183 (94, 89)^a^	71.6 overall 72.6 (i) 70.5 (c)	61.8% overall 57.5% (i) 66.3% (c)	Increased satisfaction with social support over a five year period.

**QUASI-EXPERIMENTAL STUDIES (n = 16):**				

Arnetz & Theorell [60]	60 (30, 30)	Overall NR 77.6 yrs (i) 78.8 yrs (c)	Overall NR 66.7% (i) 66.7% (c)	Increased participation in activities arranged by the bureau or occupational therapy at six months, but no effect on activities arranged by the church.

Baumgarten et al [61]	95 (51, 44)^a^	Overall NR 56.8% ≥75 yrs (i) 54.6% ≥75 yrs (c)	Overall NR 74.5% (i) 70.5% (c)	No effect on number of social ties or satisfaction with social support at 16 weeks.

Evans & Jaureguy [73]	84 (42, 42)	61.7 overall	Not reported	Reduced loneliness and increased number of social activities at eight weeks.

Fujiwara et al [74]	141 (67, 74)	Overall NR 68.2 (i) 68.7 (c)	Overall NR 77.6 (i) 68.9% (c)	At nine months, increased contact with grandchildren and children contacted via voluntary activity, and increased numbers of distant friends. Reduced support received from friends/neighbours, but increased support given to friends/neighbours. Increased number of children contacted via voluntary activity remained at 21 months.

Martina & Stevens [75]	115 (60, 55)	63.0 overall 63.2 (i) 63.1 (c)	100% overall	Six month post-intervention, more new friendships formed and improved positive and negative affect, but no effect on loneliness.

Rosen & Rosen [76]	121 (i = 68, c1 = 31, c2 = 22)	70 overall	81% overall	Increased number of new/old activities enjoyed, but no effect on social isolation or number of social events attended at 12-15 months.

Stevens & van Tilburg [77]	64 (32, 32)	Overall NR 63.4 (i) 69.8 (c)	100% overall	Trend towards reduced loneliness at one year (p = 0.054).

Toseland et al [53]	175 (i1 = 67, i2 = 51, c = 36)	Overall NR 51.7 (i1) 50.5 (i2) 50.5 (c)	100% overall	Increased support network size for support group participants at eight weeks, compared with individual counselling participants and controls. No effect on extent of support.

White et al 1999 [62]	27 (19, 8)	Overall NR 77 (i) 80 (c)	Overall NR 84 (i) 75 (c)	No effect on loneliness or social support at five months.

Winningham & Pike [63]	73 (i & c not reported)	82.1 overall	Not reported	No effect on loneliness at three months, though it maintained participants perception of their social support compared to a deterioration in the control group

Bogat & Jason [78]	35 (i1 = 12, i2 = 11, c = 12)^a^	Not reported	Not reported	At three months, both intervention groups reported increased desired network size but no effect on current network size, number of telephone calls or visits per week.

Fokkema & Knipscheer [79]	29 (15, 14)	Overall NR 66 (i) 68 (c)	Overall NR 92% (i) 50% (c)	At three years, reduced overall loneliness but no effect on social or emotional loneliness.

Mulligan & Bennett [80]	23 (i & c not reported)	77 overall 75 (i) 80 (c)	91% overall	Unable to assess intervention effect as only within-group analysis conducted.

Rook & Sorkin [81]	180 (i = 52, c1 = 69, c2 = 59)	70.5 overall 69.6 (i) 68.9 (c1) 73.2 (c2)	65.6 overall 67.3 (i) 69.6 (c1) 59.3 (c2)	No effect on loneliness or the number of people depending on participants, at one and two years compared with both control groups. Increased number of new relationships formed at one and two years, and increased number of new social ties at two years compared with both control groups.

Toseland & Smith [54]	99 (59 - not split by i1 & i2, 40)	Overall NR 50.4 (i1) 50.0 (i2) 50.5 (c)	100% overall	Neither peer nor professional counselling groups reported any effect on network size, change in support network or satisfaction with support network at eight weeks.

Bergman-Evans [65]	35 (21, 13)^a^	Overall NR 76.1 (i) 83.1 (c)	Overall NR 38.1% (i) 84.6% (c)	No effect on loneliness at one year.

a. Number in main analysis

**Table 6:** Vote counting stratified by (i) delivery mode, (ii) degree of participation and (iii) intervention type

Study	Delivery mode	Participatory/non-participatory	Intervention type	Theory-based	Social health				Mental health		Physical health
					**Loneliness**	**Social isolation**	**Structural social support**	**Functional social support**	**Depression**	**Mental wellbeing**	

Harris & Bodden [67]	Group	Participatory	Activity	Yes	-	-	*	-	-	-	-

Routasalo et al [13]	Group	Participatory	Activity	Yes	-	-	*	-	-	-	-

Fujiwara et al [74]	Group	Participatory	Activity	Yes	-	-	*	ns	-	-	*

Ollonqvist et al [70]	Group	Participatory	Activity	No	ns	-	ns	-	*	-	-

Arnetz & Theorell [60]	Group	Participatory	Activity	No	-	-	*	-	ns	ns	-

Baumgarten et al [61]	Group	Participatory	Activity	No	-	-	ns	ns	ns	-	-

Lokk [69]	Group	Participatory	Support	Yes	ns	-	*	*	*	*	*

Martina & Stevens [75]	Group	Participatory	Support	Yes	ns	-	*	-	-	*	-

Stevens & van Tilburg [77]	Group	Participatory	Support	Yes	ns	-	-	-	-	-	-

Fukui et al [55]	Group	Participatory	Support	Yes	*	-	*	*	-	-	-

Evans & Jaureguy [73]	Group	Participatory	Support	Yes	*	-	*	-	ns	-	-

Kremers et al [68]	Group	Participatory	Support	Yes	*	-	-	-	-	*	-

Toseland et al [53]	Group	Participatory	Support	Yes	-	-	*	ns	-	*	-

Winningham & Pike [63]	Group	Participatory	Support	No	ns	-	-	*	-	-	-

Savelkoul & de Witte [56]	Group	Participatory	Support	No	ns	-	-	-	-	*	*

Rosen & Rosen [76]	Group	Participatory	Support	No	-	ns	*	-	-	ns	-

Constantino [66]	Group	Participatory	Support	No	-	-	*	-	*	-	-

White et al, 2002 [64]	Group	Non-participatory	Internet training	No	ns	-	ns	-	ns	-	-

White et al, 1999 [62]	Group	Non-participatory	Internet training	No	ns	-	-	ns	ns	ns	-

Drentea et al [52]	Mixed mode	Participatory	Support	No	-	-	-	*	-	-	-

Rook & Sorkin [81]	One-to-one	Participatory	Activity	Yes	ns	-	*	ns	ns	ns	-

Brennan et al [51]	One-to-one	Participatory	Support	Yes	-	ns	-	-	ns	-	ns

Toseland & Smith [54]	One-to-one	Participatory	Support	Yes	-	-	ns	ns	-	*	-

Heller et al [58]	One-to-one	Participatory	Support	No	ns	-	-	ns	ns	ns	-

Schulz [59]	One-to-one	Non-participatory	Home visiting	Yes	-	-	*	-	-	*	*

MacIntyre et al [71]	One-to-one	Non-participatory	Home visiting	No	-	-	*	ns	-	-	-

O'Loughlin et al [57]	One-to-one	Non-participatory	Home visiting	No	-	-	ns	ns	-	-	-

Bogat & Jason [78]	One-to-one	Non-participatory	Home visiting	No	-	-	*	-	-	-	-

Mulligan & Bennett [80]	One-to-one	Non-participatory	Home visiting	No	-	ns	-	-	-	ns	-

Slegers et al [72]	One-to-one	Non-participatory	Internet training	No	ns	-	ns	-	ns	ns	-

Fokkema & Knipscheer [79]	One-to-one	Non-participatory	Internet training	No	*	-	-	-	-	-	-

Bergman-Evans [65]	Service provision	Participatory	Service provision	Yes	ns	-	-	-	*	-	-

Key: '*' = statistically significant (p < 0.05) between-group difference, 'ns' = no statistically significant (p ≥ 0.05) between-group difference, '-' = not measured

#### Group interventions providing activities

##### Moderate risk of bias

Of the three studies at moderate risk of bias [13,61,70], two reported significant intervention effects in the extracted outcomes. Community-dwelling participants of a psychosocial activity group [13] reported developing more new friendships in comparison with control participants at 12 months. Participants in a physical activity programme within an inpatient geriatric rehabilitation setting [70] reported reduced depression at 12 month follow-up, while no effect was observed on loneliness or the number of friends and relatives.

##### High risk of bias

All three studies at high risk of bias reported improvements in the extracted outcomes. An activity programme for senior citizen apartment residents [60] reportedly increased the number of participants' activities at six months, although no reduction was observed in level of depression or suicidal thoughts. However, the authors only reported intervention effects that were consistent across the six month study period; hence it is possible that short-term effects assessed at three months were missed. An evaluation of an activity group for socially disengaged community-dwelling older people [67] reported that participants increased their social interaction in comparison with the control group at six weeks. The small sample size used in this study and the lack of a longer-term follow-up period limit the interpretation of findings. Community-dwelling older people who volunteered to read books to school children [74] unsurprisingly reported increased frequency of communication with children contacted through volunteer activities at nine months. Participants also reported increased contact with grandchildren and distant friends, and improved self-rated health compared with controls. While the quantity of and amount of contact with friends or neighbours did not change, the level of social support received from them reduced and the level of social support provided by participants increased. No intervention effect was reported regarding level of support received from or provided to family members. Follow-up assessments at 21 months were restricted to volunteers (37/67) participating in "more than a few sessions every month", and to two outcomes of self-rated health and frequency of communication with children contacted through volunteer activities. Significant between-group differences favouring the intervention were observed for both outcomes, but these must be interpreted with caution due to the selective nature of participants followed up.

#### Group interventions providing support

##### Moderate risk of bias

All three studies at moderate risk of bias reported significant intervention effects. A coping group intervention [56] for people with chronic rheumatic disorders reported improved functional health status in comparison with a waiting list control group, and improved action-directed coping in comparison with a mutual support control group at 13 weeks. No intervention effect was observed between the coping group and either control group regarding loneliness at either time point. However, the results have limited generalisability to the general population of older people with chronic conditions due to the mean (SD) age of study participants (Table 5). Women with breast cancer who participated in a psychosocial group intervention [55] reported having more confidants available to them, better satisfaction with social support and reduced loneliness in comparison with the control group at six weeks and six months. The ability to draw firm conclusions from the study is however limited by the small sample size. In the third study, community-dwelling widows were randomised either to a bereavement support group, a social adjustment intervention or a control group [66]. The bereavement support group reported enhanced socialisation and reduced depression compared with the other groups. Follow-up assessments were conducted at six weeks and three, nine and 12 months. The effect on both outcomes was most notable at six weeks and subsequently reduced over time, although bereavement support group scores remained better than those of both other groups. The results suggest that the intervention may have short, rather than long-term benefits.

##### High risk of bias

Of the eight studies at high risk of bias [53,63,68,69,73,75-77], only one study [77] did not report significant intervention effects in one or more of the extracted outcome domains. Community-dwelling handicapped older people who participated in a discussion group [69] undertook follow-up assessments at six, 12 and 24 weeks. Participants reported increased social network size at six weeks and increased availability of contacts at 24 weeks, although more participants reported feeling 'often lonely' compared with controls at six weeks. Participants reported decreased depression at 24 weeks, and reduced feelings of hopelessness at all time points in comparison with the control group. Beneficial intervention effects were observed in perceived health at 12 and 24 weeks. An evaluation of an educational friendship programme for older women [75] reported that, at six months, participants developed more new friendships in comparison with a control group and demonstrated improved positive and negative affect. There was no observed reduction in participants' loneliness, no improvement in assertiveness and a non-significant trend towards improved self-esteem.

A mental health counselling group was offered to members of a senior citizen centre with evidence of depression, recent trauma or senility [76]. Participants enjoyed more new activities and rediscovered old activities when compared with an untreated comparison group at post-intervention. The authors reported no reduction in social isolation and non-significant trends suggesting a protective effect on perceived health and morale compared with controls at follow-up. A telephone-based group therapy that taught older people how to cope with their blindness [73] was reported to increase numbers of social activities and reduce levels of loneliness amongst participants in comparison with controls at eight weeks. No effect was observed on depressive symptoms. A cognitive behavioural therapy for nursing home residents [63] aimed to develop social networks and improve social interactions. At three months, participants' perception of their social support remained constant compared with deterioration in the control group, while no effect was observed on loneliness. Adult daughters and daughters-in-law who were primary caregivers for a relative with multiple chronic disabilities were allocated to one of two interventions - a support group or individual counselling - or to a control group [53]. Both interventions targeted improved coping mechanisms. At eight weeks, support group participants reported an increased support network size in comparison with controls, though no effect was observed on the extent of support, on wellbeing or on the numbers of anxiety and depression symptoms. At the same time point, beneficial effects for those receiving individual counselling were restricted to the number of anxiety and depression symptoms. The only significant difference between both intervention groups related to increased support network size at eight weeks, in favour of the support group.

A self-management group for single older women [68] aimed to reduce loneliness, focusing also on the components of social and emotional loneliness. Social loneliness refers to the perceived lack of social interactions, while emotional loneliness refers to the perceived absence of an intimate figure. No intervention effect was observed on participants' overall or emotional loneliness. Participants did however report improved social loneliness and psychological wellbeing at six weeks, although the effects disappeared at six months. However, it was unclear whether the authors were reporting between-group or within-group results; hence the data should be interpreted cautiously.

#### Group interventions providing internet training

Two studies reported internet training interventions delivered within group settings; one of which was at moderate risk of bias [64] and one was judged to be at high risk of bias [62]. Neither study reported any significant intervention effects on the outcomes extracted.

#### One-to-one interventions providing activities

##### High risk of bias

The only study evaluating a one-to-one intervention that provided social activity [81] was deemed to be at high risk of bias. Volunteers participated in a foster grandparent programme for developmentally-disabled children. At the one-year and two-year follow-ups, intervention group participants reported forming more new relationships in the past year compared with two control groups (an alternative type of group programme and a no intervention group). The intervention group reported more new social ties at the two-year follow-up in comparison with both control groups. Despite the observed beneficial effects on structural social support, there were no significant between-group differences regarding loneliness, depression or self-esteem at either time point. Additionally, no intervention effect was evident at either follow-up in respect of the number of people that depended on the participants. The authors noted that their findings had limited generalisability due to a large attrition rate (81/180, 45%).

#### One-to-one interventions providing support

##### High risk of bias

While there was one study judged to be at moderate risk of bias [51], it did not report significant intervention effects in the participant outcomes extracted. Of the two studies at high risk of bias [54,58], one reported significant intervention effects. Individual counselling, provided either by professionals or peers [54], was evaluated for use with adult daughters and daughters-in-law who were the primary caregiver for a relative suffering from multiple chronic disabilities. At eight weeks, compared with the control group, participants receiving professional counselling reported improved wellbeing and reduced psychiatric symptoms, according to an overall score and the three subscales of depression, anxiety and hostility. At the same time point, those receiving peer counselling reported only reduced psychiatric symptoms in respect of the overall score and the anxiety subscale, in comparison with controls. Neither intervention group reported improved network size or level of available support compared with the control group. No significant differences were observed between the professional and peer counselling groups on any outcomes.

#### One-to-one interventions providing internet training

##### High risk of bias

One study was at moderate risk of bias [72], although it did not report any significant intervention effects in the extracted outcomes. The only study at high risk of bias concerned an internet training intervention for community-dwelling older people who lived alone, who were housebound through chronic illness or physical disability, and who were current participants in a home visiting scheme [79]. At the three-year follow-up, intervention group participants reported a significantly greater reduction in overall loneliness, in comparison with the control group, while no difference was observed on the sub-scales of social and emotional loneliness.

#### One-to-one interventions providing home visiting

##### Moderate risk of bias

Of the two studies at moderate risk of bias [57,71], only one reported significant intervention effects. A volunteer home visiting intervention was offered to community-dwelling older people in receipt of home nursing, who were considered by their nurses to be socially isolated or lonely [71]. There was some evidence of improved social support at six weeks, as participants reported increased social integration and feelings of worth. However, no intervention effect was observed on the social support domains of intimacy, nurturance and guidance at the same time point.

##### High risk of bias

Two of the three studies at high risk of bias [59,78,80] reported significant improvements in the extracted outcomes. Community-dwelling older people who were on a waiting list for a friendly visiting programme were offered either a network-building or a relationship-orientated visiting programme [78]. At three month follow-up, participants in both programmes reported having a desire for larger social networks in comparison with the control group, though this did not lead to larger current social networks, or a greater number of visits or visitors, or phone calls per week. The second study [59] evaluated whether predictability and control influenced the effectiveness of a home visiting intervention for retirement home residents. At two months, in comparison with the control groups (receiving random visits and no visits), intervention participants (controlling frequency/duration of visits, and advance notice of visits) reported more activity, a greater amount of time spent in active pursuits and planned more activities for the coming week. The intervention groups also reported improved hope and happiness, and reported a smaller increase in the quantity of medication taken per day in comparison with control participants. No significant differences were observed between the two intervention groups, or between the two control groups.

#### Mixed mode interventions

##### High risk of bias

The only study of a mixed mode intervention concerned counselling for older people who were caregivers to Alzheimers' disease sufferers [52], with counselling delivered at both the individual and group level. Regression analysis revealed that, post-intervention (four months) the intervention group reported higher satisfaction with social support in comparison with the control group. The observed effect remained constant over the five year study period in which intervention group participants could attend support groups and contact counsellors.

#### Service provision interventions

##### High risk of bias

The only study that assessed service provision was an evaluation of an alternative form of nursing home care, whereby nursing home residents had daily contact with children, pets and plants [65]. Study participants reported reduced helplessness and boredom, but no reduction in loneliness at the one-year follow-up. Contrary to the authors' claims, there appeared to be large between-group differences in outcome scores at baseline, with a higher proportion of the control group being classified as lonely, helpless and bored. It is not clear whether the analyses were adjusted for the apparent baseline differences, meaning that the results should be interpreted cautiously.

#### Intervention effects according to intervention characteristics

Table 6 summarises the study results relating to the three outcome domains extracted, stratified by delivery mode (group, one-to-one, mixed mode, service provision), whether or not the intervention is participatory, and the presence of a theoretical basis. The lack of studies representing mixed (n = 1) and service provision (n = 1) delivery modes precluded them from this analysis.

Using count data (Table 6) to analyse delivery mode (group-based and one-to-one) indicated a disparity in their apparent effect. Thirteen out of 19 (68%) group interventions in this review [13,53,55,60,63,66-69,73-76] had a positive effect on at least one of the four social health sub-domains. This compares with five out of 11 (45%) one-to-one interventions [59,71,78,79,81]. The disparity between the two delivery modes remains when including evidence from the mental health sub-domains of 'depression' and 'mental or psychological wellbeing' and from the physical health domain. The proportion of effective group interventions rose to 79% (15 out of 19) with the addition of two studies [56,70], and the proportion of effective one-to-one interventions rose to 55% (6 out of 11) with the addition of one study [54]. Thus, group-based activities appear more effective across a wider range of outcome domains compared with those offered on a one-to-one basis.

Comparing the effectiveness of participatory and non-participatory interventions across the three domains also indicated differential effects. Nineteen out of the 23 (83%) participatory interventions were observed to have beneficial effects on at least one outcome [13,52-56,60,63,65-70,73-76,81] compared with only four out of nine (44%) non-participatory interventions [59,71,78,79].

Another characteristic related to the effectiveness of interventions was the presence of a clear theoretical base (Table 6). Thirteen out of 15 (87%) interventions categorised as having a theoretical basis reported beneficial effects on at least one outcome domain [13,53-55,59,65,67-69,73-75,81] compared with 10/17 (59%) studies categorised as having no theoretical basis [52,56,60,63,66,70,71,76,78,79].

Interventions that explicitly targeted people who were socially isolated or lonely (not shown in Table 6) were less likely to produce beneficial effects across all domains. Seven out of 12 studies (58%) that recruited explicitly targeted participants reported positive effects on outcomes [13,56,67,71,75,78,79], compared with 16/20 (80%) studies with no explicit targeting [52-55,59,60,63,65,66,68-70,73,74,76,81].

Regarding the type of intervention provided (Table 6), six out of the seven (86%) activity interventions had at least one beneficial effect across the three domains of social, mental and physical health [13,60,67,70,74,81]. Twelve out of the 15 (80%) support interventions reported beneficial effects [52-56,63,66,68,69,73,75,76]. Three out of five (60%) home visiting interventions led to beneficial effects [59,71,78], as did one of the four (25%) interventions offering internet training [79]. The remaining intervention concerned service provision [65], which reported beneficial effects.

The proportion of studies reporting positive effects across the three outcome domains also appeared to differ according to the nature of the intervention provider (not shown in Table 6). All interventions delivered by counsellors [53,54,73] and by students [59,66,71,78] reported at least one beneficial effect, compared with nine out of 11 (82%) interventions delivered by health and social care professionals [13,52,56,60,65,69,70,76,81]. Six studies provided limited information about the background of personnel delivering the intervention (e.g. female leader, professional, trained interviewer), three of which (75%) reported positive treatment effects [55,68,74]. One out of the four (25%) interventions delivered by IT instructors led to beneficial effects [79]. Four studies provided no information about the person providing the intervention, three of which (75%) reported beneficial outcomes [63,67,75].

## Discussion

The likelihood of interventions producing beneficial effects may differ according to their characteristics. For example, those offered at a group level were more likely to be beneficial compared with one-to-one interventions, and those defined as being theoretically-based tended to be more beneficial than those that were not. Participatory interventions and those including social activity and support were also more likely to be beneficial. While the nature of the intervention provider appeared to be a factor on the basis of vote counting, this should be interpreted cautiously due to the large number of providers identified and the small number of studies relating to each one. There are indications that social isolation interventions may have wide-ranging benefits including structural social support, functional social support, loneliness, and mental and physical health. This study advances the evidence base of previous reviews [5,40,41], by including studies published since 2002 and by considering a wider range of outcomes reflecting the multi-dimensional definition of social isolation.

Possible explanations for the variability of effects according to intervention characteristics include the content of interventions and the methodological quality of studies. The interventions may have been poorly developed, lacking appropriate theoretical basis and subsequent components to impact on the measured outcome domains [82,83]. Limitations with study design (e.g. small sample sizes, high attrition rates) or data analysis may also have caused the true intervention effects to be missed or over estimated due to confounding variables [84].

Despite the included studies evaluating interventions aiming to alleviate social isolation or loneliness, only 12 out of 32 (38%) studies explicitly targeted people in this situation. In the remaining 20 studies, the underlying 'baseline risk' [85] of social isolation or loneliness was implicitly assumed due to other characteristics of the specific client group, such as being a resident in a nursing home. If some or all study participants do not have the problem being addressed by an intervention at baseline, the true treatment effect may be masked. One explanation for this apparent lack of targeting may be the difficulty of measuring the concept of social isolation. However, data from our review indicated that interventions targeting older people identified as being socially isolated or lonely were no more likely to result in beneficial effects across the outcome domains synthesised.

### Strengths and weaknesses of the review

The use of a comprehensive search strategy and the inclusion of both randomised and quasi-experimental trial designs increased the number of papers reviewed, optimising our likelihood of including all relevant studies. Extracting data for a greater number of outcomes than in previous reviews allowed us to assess the wider effectiveness of interventions for reducing social isolation, as well as to look for evidence regarding their potential health benefits

The lack of consensus regarding the definition of social isolation had implications for both the identification of studies and the interpretation of reported findings. While we made considerable effort to ensure the robustness of our search strategy, potentially relevant studies may not have been identified if authors did not use terms that we included. Had we adopted a uni-dimensional definition of social isolation (e.g. a lack of social integration) we may not have extracted functional social support outcome data; the absence of which may have influenced interpretation of the count data.

Limiting study eligibility to those published in English may also have introduced bias [86], although this decision was made for pragmatic reasons. While the date range of our search period enabled us to identify studies published in the seven years following the most recent systematic reviews [5,40], this is a rapidly emerging field. For example, since the end of our search period (May 2009), new analysis [87] of data derived from a study already included in our review [13] has provided additional evidence suggesting beneficial effects for a psychosocial group intervention.

Comparability of study findings was limited by the heterogeneity of evaluated interventions, and the participants and outcomes assessed. The poor reporting and poor quality of many of the studies was a major limitation of the literature. Our decision to jointly present findings of randomised and non-randomised studies may also be considered a limitation. While we acknowledge that non-randomised studies are at increased risk of bias, there is an argument that poor quality RCTs may be lower value than well-conducted non-randomised controlled trials [88,89]. In light of this, the absence of high quality studies of either design justifies our approach as it enables data to be discussed concisely. In addition, the majority of studies were conducted in either Scandinavia (n = 4), the Netherlands (n = 6) or the United States (n = 17), which limits generalisability to other countries. The organisation of statutory and voluntary services differs between countries; hence the comparability of control groups is particularly difficult as 'usual care' may be country-specific.

### Implications for targeting social isolation in older people

While interventions targeting social isolation include some of the beneficial characteristics identified in this and previous systematic reviews [5,40], none appear to include *all *of them. We suggest that developing interventions with this in mind may optimise their likelihood of success. It is also necessary for the quality of conduct and reporting of evaluations to improve, to provide better evidence. While experimental study designs are not always feasible or acceptable [90,91], efforts to use randomisation where possible should be encouraged [46]. Increased adherence to reporting guidelines such as CONSORT [92] for RCT studies and STROBE [93] for observational studies will also enhance the utility of intervention evaluations. The inclusion of rigorous process evaluations within trials may also shed light on the mechanisms through which social isolation may be reduced [94].

## Conclusions

Our systematic review has identified a need for well-conducted studies to improve the evidence base regarding the effectiveness of social interventions for alleviating social isolation. However, it appeared that common characteristics of effective interventions may include having a theoretical basis, and offering social activity and/or support within a group format. Interventions in which older people are active participants also appeared more likely to be effective.

## Competing interests

The authors declare that they have no competing interests.

## Authors' contributions

AD developed the systematic review, with comments on the protocol and search strategy from JC, SR and CG. AD conducted the searches and data extraction, with SR and CG acting as second reviewer at different stages. AD conducted the narrative synthesis of the extracted data and wrote the initial draft, with JC, SR and CG contributing to revised drafts. All authors read and approved the final manuscript.

## Pre-publication history

The pre-publication history for this paper can be accessed here:

http://www.biomedcentral.com/1471-2458/11/647/prepub
